# Oil Lamp Dangers

**Published:** 1874-07

**Authors:** 


### OIL LAMP DANGERS.

BEING burned to death is the most terrible of all human sufferings, and yet that is the fate of quite a number every year from the want of proper precautions.

NEVER FILL A LAMP IN THE HOUSE.

NEVER FILL THE LAMP AFTER DARK.

NEVER REPLENISH A LAMP WHILE BURNING, WHETHER BY DAY OR NIGHT.

IN HANDLING OIL, LET IT BE TEN FEET AWAY FROM ANY FIRE OR FLAME.

The first thing to be done after purchasing the oil and bringing it home is to ascertain whether it is dangerous, thus : fill a saucer half full of water, pour on the surface of it a tablespoonful of the oil ; apply a burning paper to the oil ; if it catches in a flame it is not safe. If a glass lamp is broken while kerosene oil is burned in it, and the oil takes fire, such oil should not be used ; it is not necessary that such oil should be used, for it is bad, impure kerosene.

Terrible accidents have occurred from kindling a fire by pouring kerosene on the wood after the match has been applied to the kindling. Pour the oil on first, two or three teaspoonfuls only, close the can and remove it to a distance, and then apply the match. As millions of property and many lives are lost every year from kerosene accidents, as they are called, and which could be easily avoided, it becomes the duty of all to inform themselves, and to impart information to those under them as to the safe way of handling all inflammable mixtures.

After all, they are wise who will not allow a drop of kerosene oil inside their dwellings, any more than a drop of spirits ; the latter, however, is the most dangerous and the most dreadful in its effects.

